# Increasing use of linezolid in a tertiary NICU during a 10-year period: reasons and concerns for the future

**DOI:** 10.1186/s13756-020-00818-2

**Published:** 2020-09-23

**Authors:** Lucie Matrat, Frank Plaisant, Christine Barreto, Olivier Claris, Marine Butin

**Affiliations:** 1 Service de Néonatologie et Réanimation Néonatale, Hôpital Femme Mère Enfant, Hospices Civils de Lyon, Bron, France; 2 Département d’Hygiène Hospitalière, Groupement Hospitalier Est, Hospices Civils de Lyon, Bron, France; 3 EA 4129, Université Claude Bernard, Villeurbanne, France; 4 CIRI, Centre International de Recherche en Infectiologie, Inserm U1111; CNRS UMR5308; Ecole Normale Supérieure de Lyon; Université Lyon 1, Lyon, France

**Keywords:** Linezolid, Vancomycin, NICU, Late-onset sepsis, Multidrug resistance

## Abstract

**Background:**

Linezolid has been increasingly used in tertiary NICUs. The objectives of this study were to explore the indications of these linezolid prescriptions, to analyze a possible misuse and to provide solutions to avoid such misuse.

**Methods:**

A monocentric retrospective cohort study included all neonates hospitalized in one tertiary NICU between January 1st, 2010 and December 31st, 2019 and who received at least one administration of linezolid. These data were confronted to epidemiological and antibiotic use data from the same NICU. Two independent pediatricians secondarily classified linezolid uses as adequate or not.

**Results:**

During the study period, 66 infections in 57 patients led to linezolid use. Most patients were pre-term and 21 patients (37%) died. Infections were mainly related to methicillin-resistant coagulase negative staphylococci and were frequently either pneumoniae (35%) or isolated bacteremia (48%), including 25 persistent bacteremia (64% of the 39 bacteremia). Need for a better tissue distribution or first-line treatment failure were the main reasons to initiate linezolid. Linezolid was administered for a median duration of 7 [3;10] days. No side effects were reported. Twenty-two (33%) of the 66 linezolid prescriptions were retrospectively classified as inadequate.

**Conclusions:**

A rapid increase in linezolid prescriptions has been observed in our tertiary NICU, from 2014 to 2019, with 33% inadequate uses. This worrisome trend should lead to search for therapeutic alternatives and to work on antibiotic stewardship to prevent the emergence of new antimicrobial bacterial resistance.

## Background

At birth, neonates can be hospitalized in neonatal intensive care units (NICUs) for a variety of reasons, prematurity being the leading one. With advances in neonatal care over the past decades, the survival of these vulnerable neonates has increased, but so did the use of long-term invasive procedures and devices, exposing neonates to an increasing risk of nosocomial infections [1]. The lack of specificity of septic signs in neonates and the high frequency of nosocomial sepsis in NICUs lead to wide antibiotic indications (with rapid de-escalation if the diagnosis is refuted) [2]. Because the bacteria that are the most frequently involved in these infections are Gram positive bacteria including by order of frequency methicillin-resistant coagulase-negative staphylococci (CoNS), *Staphylococcus aureus* and *Enterococcus* [2–4], vancomycin is usually prescribed as a first-line antibiotic in NICUs.

Linezolid is an alternative antibiotic belonging to the oxazolidinone class, active against methicillin-resistant Gram positive bacteria and is approved for clinical use since the early 2000s. In neonates, linezolid seems to constitute a good alternative to vancomycin because of its wide bacterial spectrum, its ubiquitous tissue distribution and its excellent bioavailability allowing for an oral administration [5–9]. In the literature we observe a constant and rapid increase of the articles reporting its use in neonates. Moreover some NICUs have reported on the increasing use of this antibiotic at the local scale [10, 11]. Such an increase has also been observed in our NICU setting in Lyon, France over the past decade.

We hypothesized that this increase in the use of linezolid in neonates in our NICU setting could be related to the emergence of vancomycin-resistant bacteria. But we cannot exclude an inadequate use of this antibiotic. Therefore, the purpose of the present study was to describe the use of linezolid in one tertiary NICU setting during a 10-year period. We then discussed indications of prescription and raised concerns about its tolerance and inadequate overprescription.

## Methods

### Study design and eligibility criteria

This monocentric retrospective cohort study was conducted in the tertiary NICU of the Hôpital Femme Mère Enfant, Hospices Civils de Lyon, Lyon, France. All neonates hospitalized in this NICU between 1st January 2010 and 31st December 2019 for whom at least one administration of linezolid was encoded in the medical record were eligible for inclusion. Patients for whom linezolid was previously initiated in another NICU before referral to the study NICU were not included.

### Collection of clinical data

The list of patients eligible for inclusion and the clinical data were retrieved using the software ICCA (Philips®, Suresne, France) which is used to prospectively record medical information for NICU patients. For each included patient the following items were collected: date of hospitalization in the study NICU, delivery mode, weight and gestational age at birth, sex, Apgar score, death during hospitalization in the NICU, data about the infection leading to the use of linezolid including age at the onset of the sepsis, infective agent, infection site, need for central line removal, and finally data about the antibiotic treatment including first-line treatment, second-line treatment, delay until the introduction of linezolid, duration and route of linezolid administration, reason to stop it, reported side effects notably thrombopenia, anemia, hyperlactacidemia. Moreover, data about the strains involved in these infections were obtained from the laboratory record, including antimicrobial susceptibility tests. According to EUCAST recommendations, resistance to vancomycin was defined as a minimal inhibitory concentration > 2 mg/L for *S. aureus* or > 4 mg/L for CoNS and *Enterococcus*. Resistance to linezolid was defined as a minimal inhibitory concentration > 4 mg/L.

### Classification of adequate/inadequate indication of linezolid

The administration of linezolid was secondly classified as adequate or inadequate independently by two pediatricians, following previously published Australian guidelines [12]. Briefly, indication was considered as adequate if linezolid was prescribed for a Gram positive infection with either resistance to other antibiotics (including beta-lactams and vancomycin), or if there was a persistent bacteremia despite vancomycin therapy with appropriate serum level (15–20 mg/L) and removal of central venous line (or impossibility to remove it), or also if there was a contraindication to vancomycin (renal failure). In other situations, linezolid was considered as inadequate.

### Data about other antibiotics

To have an overview of all the antibiotics used during the study period in this NICU and especially antibiotics targeting Gram positive bacteria, we extracted from the software ICCA the number of patients that have received at least one course of vancomycin, rifampicin, fosfomycin, oxacillin and cefazolin. The use of each antibiotics was expressed as number of patients receiving at least one course of each antibiotic for 1000 patient-days.

### Microbiological and epidemiological data

To confront our data with the microbial epidemiology of infections during the study period, all bacterial bloodstream infections that occurred in this NICU during the study period were retrieved from the epidemiological monitoring routinely performed by the Department of Infection Control of the hospital and were expressed as number of septic episode for 1000 patient-days. Of note in this NICU, clinical reports of all patients with a positive blood culture are routinely analyzed by both a NICU practitioner and a medical officer of health, to classify positive blood cultures as bloodstream infections or contaminant. Bloodstream infection was defined as the association of clinical signs with at least one positive blood culture. Pneumonia was defined as the combination of physical exam findings, radiographic evidence, and a positive culture of suctioned tracheal sputum. Persistent bloodstream infection was defined as more than 2 blood cultures positive for a same bacterium and spaced out of at least 48 h.

### Ethics

This monocentric retrospective cohort study was approved by the local institutional ethics committee (Hospices Civils de Lyon) under the number 20–62 and was registered to the French data protection authority under the number 20–137. A written information about this study was delivered to the families of the included patients to give them opportunity to decline participation to this study. In accordance with French legislation, a written consent was not required owing to the observational nature of the study. All clinical data were anonymized.

### Statistics

A descriptive analysis was performed. Patient characteristics were described as median, interquartile range (IQR) or as percentage. No statistical test was performed for this observational study.

## Results

### Antibiotic use and bacterial epidemiology

During the 10-year study period, linezolid was prescribed 67 times in 58 patients in this NICU but one patient was not included because linezolid therapy was already started at his arrival in the study center. Thus, the analysis was performed on 66 linezolid prescriptions in 57 patients.

**Fig. 1:**
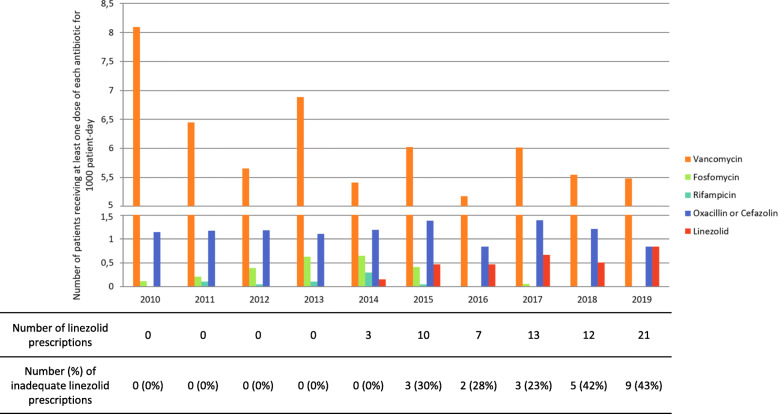
Antibiotic use in the study NICU (2010–2019)

The frequency of linezolid prescription rapidly increased since no prescription was recorded before 2014 and more than 10 per year (more than 0.5 patients for 1000 patient-days) after 2017 (Fig. 1). During the same period, vancomycin and oxacillin/cefazolin prescriptions were stable. In parallel, the prescriptions of the two alternatives frequently prescribed for methicillin-resistant CoNS, fosfomycin and rifampicin sharply decreased after 2015.

**Fig. 2:**
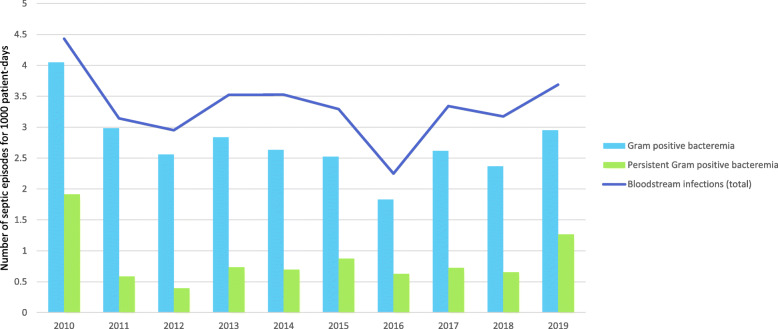
Epidemiology of bloodstream infections, including Gram positive bacteremia and persistent bacteremia in the study NICU (2010–2019)

This increase in linezolid prescription was not associated with an increase in bloodstream infections especially those involving Gram positive bacteria, nor with an increase in persistent bacteremia (Fig. 2).

### Patients characteristics

The characteristics of the 57 patients of this study are presented in Table 1. Among the 57 patients, most were pre-term (*n* = 54, 95%), with a median gestational age at birth of 26 gestational weeks (IQR [25;27]) and a very low birth weight with a median weight of 717 g (IQR [590;880]). Nineteen of the 57 neonates (33%) were intubated within the first 10 min following birth, 33 neonates (58%) had Apgar score ≥ 7 at M5. Twenty-one patients (37%) died during their hospitalization in NICU including 12 deaths related to the infectious episode that led to linezolid prescription.

**Table 1:** Characteristics of the 57 neonates that received linezolid in the study NICU between 2010 and 2019

	n (%)
Sex ratio (M/F)	0,63
In born	45 (79%)
Delivery: caesarian mode	44 (77%)
Prematurity (GA <37GW)	54 (95%)
Gestational age at birth (GW)^a^	26 (25;27)
Weight at birth (g)^a^	717 (590;880)
Apgar score at M3^a^	6 (4;8)
Apgar score at M5^a^	8 (6;9)
Apgar score at M10^a^	10 (9;10)
Intubated before M10	19 (33%)
Death during hospitalization in the NICU	21 (37%)

^a^Median (interquartiles)

### Infections characteristics

Sixty-six infectious episodes led to the prescription of linezolid in these 57 neonates; the main characteristics of these episodes are presented in Table 2. These infections occurred at a median age of 20 days (IQR [9; 33]). Infections were frequently either isolated bacteremia (48%) or pneumoniae (35%). A positive blood culture was retrieved in 39 cases, including 25 persistent bacteremia. The 66 infections were mainly related to methicillin-resistant CoNS, followed by *S. aureus* and *E. faecalis*. In seven infectious episodes (11%), no bacteria was identified. Among all identified bacteria, only two strains (one *Staphylococcus epidermidis* and one *Staphylococcus capitis*) harbored a resistance to vancomycin and none was resistant to linezolid.

**Table 2:** Characteristics of the 66 infectious episodes that led to linezolid prescription in the study NICU (2010–2019)

	n (%)
**Age at first symptoms (days)**^**a**^	20 (9;33)
**Hemodynamic instability**	28 (42%)
**Strains**	
*Staphylococcus epidermidis*	23 (35%)
*Staphylococcus capitis*	11 (17%)
*Staphylococcus aureus*	9 (14%)
*Staphylococcus haemolyticus*	3 (5%)
*Enterobacter cloacae*	1 (2%)
*Staphylococcus gallinarum*	1 (2%)
No identification (clinical sepsis)	7 (11%)
Polymicrobial	11 (17%)
**Resistance to vancomycin**	2 (3%)
**Site of infection**	
All bacteremia	39 (59%)
Including persistent bacteremia	25 (64%)
Isolated bacteremia	32 (48%)
Pneumoniae	23 (35%)
Other (CSF, peritoneal fluid)	4 (6%)
No identification (clinical sepsis)	7 (11%)
**First-line treatment**	
Vancomycin	51 (77%)
Linezolid	12 (18%)
Other	3 (5%)
**Antibiotic association (≥ 2 antibiotics)**	46 (70%)
including amikacin	43 (93%)
**Delay before linezolid use (days)**^a^	3 (2;6)
**Linezolid treatment duration (days)**^a^	7 (3;10)
**Central line removal**	25 (38%)
In case of persistent bacteremia	21 (88%)

^a^Median (interquartiles)

The most frequent first-line antibiotic was vancomycin (77%) whereas linezolid was part of the first-line treatment in 12 (18%) septic episodes. In 46 (70%) episodes at least1 s antibiotic was associated. Linezolid was started at a median of 3 days (IQR [2;6]) after the onset of symptoms. The main reasons for which clinicians have chosen linezolid were, by order of frequency: tissue distribution (especially for pulmonary infection), failure of first-line treatment, contraindication to vancomycin, probabilistic antibiotic therapy, resistance to vancomycin and need for an oral route. Linezolid was administered for a median duration of 7 days (IQR [3;10]). An early stop of linezolid was observed in 25 infectious episodes either because of the death of the patient in 12 cases, or because the antibiotic regimen was switched for another in 8 cases or because the diagnosis of infection was ruled out in 5 cases. No side effects were reported in the 57 patients.

### Classification as adequate/inadequate

**Fig. 3:**
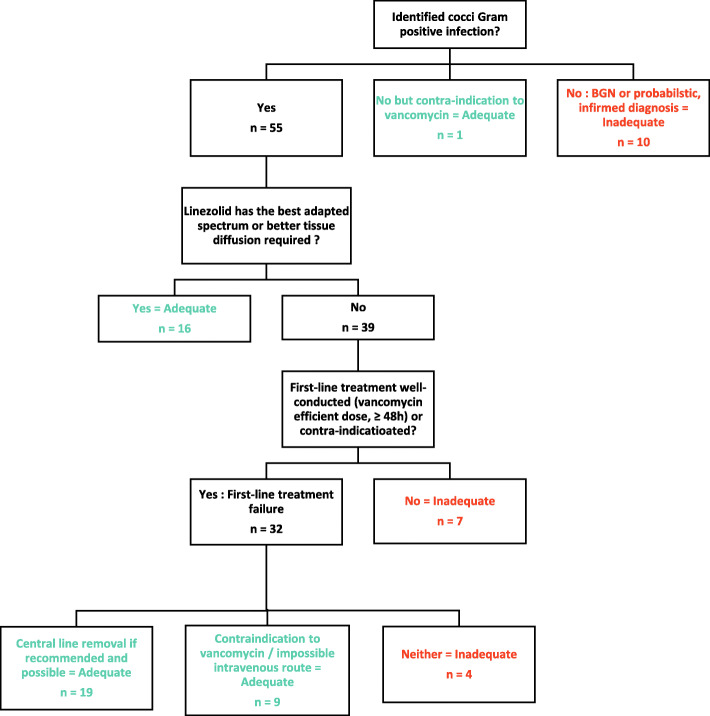
Classification by 2 pediatricians of the 66 linezolid prescriptions as adequate or inadequate following a standardized algorithm

After evaluation by two pediatricians 22 (33%) of the 66 linezolid prescriptions were classified as inadequate (Fig. 3). Of note, the proportion of inadequate prescriptions increased with time with 0% of all prescriptions classified as inadequate in 2014 versus 43% in 2019 (Fig. 1). The major causes of inadequate prescription of linezolid were either an absence of true infection (probabilistic use) without contraindication to vancomycin, or a non- optimal first-line treatment (low vancomycin serum level and/or absence of removal of central line whereas it was feasible). The most frequent situations of adequate linezolid prescription were either the failure of vancomycin first-line treatment, or a pulmonary infection requiring a correct tissue diffusion.

## Discussion

The present study illustrates a rapid and worrying increase in linezolid prescriptions in a tertiary NICU over the past decade. The retrospective analysis of the 66 linezolid prescriptions in this NICU revealed that one third of these prescriptions were considered as inadequate.

Thanks to its wide bacterial spectrum, including Gram-positive bacteria and its excellent bioavailability, linezolid emerged in the early 2000s as a good alternative to vancomycin. In the specific population of neonates, literature data dealing with linezolid pharmacology, safety, effectiveness and tolerance in neonates is exponential thus demonstrating its growing importance [6, 8, 9, 13]. In the study NICU, the first use of this drug was reported in 2014 and then increased yearly, raising our concern and leading to this work. Such increase has already been reported by other teams. For example, Buccelatto et al reported on a 3-fold increase in linezolid prescriptions from 2004 to 2011 in hospitalized children [10] while Bagga et al described a 5-fold increase in linezolid use between 2007 and 2014 in an American children’s hospital [11].

The reasons of this rapid increase are not totally understood. The hypothesis of Buccelatto et al was that it was due to an increase in glycopeptide heteroresistant or resistant staphylococci [10]. Indeed, the presence of a decreased susceptibility to vancomycin is an emerging issue in CoNS strains involved in neonatal sepsis [14, 15] and linezolid has been reported as an efficient alternative in those situations [9]. However, in our study only 2 strains harbored a documented resistance to vancomycin and no significant change in the bacterial epidemiology was noted during the study period so this hypothesis is not sufficient to explain the increase in linezolid prescriptions. Another situation in which linezolid was adequately prescribed in our study along with other ones is the need to obtain a correct pulmonary diffusion, for example in case of methicillin-resistant CoNS or *S. aureus*-related pneumonia [16]. Of note, this situation concerned 14 cases (21%) in our cohort. Additional reasons to prescribe linezolid that are brought forth by prescribers in our study included: first-line treatment failure with persistent bacteremia despite adequate treatment, contraindication to first-line treatment (notably oliguric renal insufficiency) or impossible venous access. Such situations have already been discussed in the literature and considered as adequate [17].

However, in parallel to these situations, we also noted a 33% rate of linezolid prescriptions that were classified as inadequate as well as a disquieting rapid increase of these inadequate prescriptions, reaching more than 40% both in 2018 and 2019. The major situations of inadequate use in our cohort were the prescription in first-line in absence of contraindication to vancomycin or in second-line despite a non-optimal first-line treatment (insufficient vancomycin serum level and/or absence of removal of central line whereas it was if feasible). Due to the retrospective design of our study we can only speculate about the reasons of such increasing use in this NICU. An hypothesis is that linezolid was firstly used only in 2014, so physicians were initially very cautious in its prescription but linezolid is now considered as a usual drug that can be used in first-line.

The inadequate prescription of linezolid and its constant increase are of high concerns, first because it can lead to the selection and emergence of linezolid-resistant strains in NICU settings. Fortunately, hitherto we did not notice any emergence of bacterial resistance secondary to linezolid uses in our setting. However some authors have already reported such phenomenon [18, 19]. A second concern related to the wide use of linezolid in NICUs is the possible side effects of this drug in neonates. As a matter of fact, linezolid is a relatively recent drug with limited data in neonates. Previous data in children suggest that linezolid could induce reversible myelosuppression (mostly thrombocytopenia but also anemia and leucopenia) [20], lactic acidosis [21], gut disorders [13] and in rare cases neuropathy [22]. In our cohort no side effects were reported. This suggests that these side effects are limited especially in case of short linezolid course. However, we cannot exclude an underestimation of these side effects because there was no systematic biological analysis in patients treated with linezolid in the NICU study.

Due to the high and increasing number of inadequate linezolid use, as well as the possible risks related to this use, it is urgent to propose several ways for improvement. First, linezolid should be replaced when possible by another antibiotic drug. In the situations of infection with methicillin-susceptible CoNS or *S. aureus* it is obvious that the first choice has to be a penicillin M. Similarly, in case of penicillin-susceptible *Enterococcus* or *Streptococcus*, a penicillin A has to be prescribed. For methicillin-resistant Gram-positive bacteria, the choice is more difficult. Before 2014, linezolid was never used in the study NICU and rifampicin adjuvant therapy or fosfomycin were used in case of persistent bacteremia despite optimal vancomycin administration or in nosocomial pneumonia. Those drugs can still be considered in those situations, since literature data attest of their good efficiency in such indications [23–25]. More recently, new drugs namely ceftarolin and ceftobiprole have been discovered and commercialized and can constitute very interesting alternatives. However, they have been rarely used in this population so data are lacking in the literature [26]. Along with the choice of drugs, the removal of central line is recommended in case of persistent bacteremia and should not be forgotten. Finally the control of antibiotics’ use and the presence of a referring infectious disease specialist in NICUs may help to reduce antibiotic pressure [27], using for example care bundle methods of antibiotic stewardship as Ting et al implemented in 2014 in British Columbia Women’s Hospital and Health Centre [28]. The implementation of such care bundles is currently considered in the study NICU.

Our study presents some limitations. The retrospective classification of adequate/inadequate linezolid use was in some cases difficult due to lacking and/or not standardized data in the medical record. This study being monocentric, and even if our results are consistent with the literature, we cannot generalize our findings to all NICU settings. Finally, bacterial epidemiology of nosocomial pneumonia during the study period was not available so it was not possible to know if the increasing use of linezolid could be due to an increasing number of these infections.

## Conclusions

Appropriate antibiotic use is one of the top public health priorities. A prospective and multicenter survey of broad-spectrum antibiotics prescription in NICUs might be implemented to limit the risk of emerging multidrug resistant bacteria for which alternatives are lacking especially in neonates.

## Data Availability

The datasets used and/or analyzed during the current study are available from the corresponding author on reasonable request.

## Abbreviations

CoNS: Coagulase-Negative Staphylococci
NICU: neonatal intensive care units
GW: gestational weeks
IQR: interquartile range
CSF: cerebro-spinal fluid
